# The Effect of Holder Pasteurization on Nutrients and Biologically-Active Components in Donor Human Milk: A Review

**DOI:** 10.3390/nu8080477

**Published:** 2016-08-02

**Authors:** Chiara Peila, Guido E. Moro, Enrico Bertino, Laura Cavallarin, Marzia Giribaldi, Francesca Giuliani, Francesco Cresi, Alessandra Coscia

**Affiliations:** 1 Complex Structure Neonatology Unit, Department of Public Health and Pediatric, University of Turin, Via Ventimiglia 3, Torino 10126, Italy; enrico.bertino@unito.it (E.B.); quo.g@libero.it (F.G.); fcresi@gmail.com (F.C.); alessandra.coscia@unito.it (A.C.); 2 Italian Association of Human Milk Banks, Via Libero Temolo 4, Milan 20126, Italy; guidoemoro@tiscali.it; 3 Institute of Sciences of Food Production, National Research Concil, largo Braccini 2, Grugliasco 10095, Italy; laura.cavallarin@ispa.cnr.it (L.C.); marzia.giribaldi@crea.gov.it (M.G.); 4 Food & Nutrition Research Center, Council for Agricultural Research and Economics, via Ardeatina 546, Roma 00178, Italy

**Keywords:** human milk, donor milk, holder pasteurization, Human Milk Banks

## Abstract

When a mother’s milk is unavailable, the best alternative is donor milk (DM). Milk delivered to Human Milk Banks should be pasteurized in order to inactivate the microbial agents that may be present. Currently, pasteurization, performed at 62.5 °C for 30 min (Holder Pasteurization, HoP), is recommended for this purpose in international guidelines. Several studies have been performed to investigate the effects of HoP on the properties of DM. The present paper has the aim of reviewing the published papers on this topic, and to provide a comparison of the reported variations of biologically-active DM components before and after HoP. This review was performed by searching the MEDLINE, EMBASE, CINHAL and Cochrane Library databases. Studies that clearly identified the HoP parameters and compared the same DM samples, before and after pasteurization, were focused on. A total of 44 articles satisfied the above criteria, and were therefore selected. The findings from the literature report variable results. A possible explanation for this may be the heterogeneity of the test protocols that were applied. Moreover, the present review spans more than five decades, and modern pasteurizers may be able to modify the degradation kinetics for heat-sensitive substances, compared to older ones. Overall, the data indicate that HoP affects several milk components, although it is difficult to quantify the degradation degree. However, clinical practices demonstrate that many beneficial properties of DM still persist after HoP.

## 1. Introduction

Human milk (HM) is the gold standard for the feeding and nutrition of preterm and term newborns [1,2,3]. A mother’s own milk is the first choice for improving the short- and long-term outcomes for all neonates [1]. However, the benefits of HM are mediated by several specific bioactive and immunomodulatory factors, and HM can be considered a species-specific biological “dynamic” system [4].

When a mother’s own milk is unavailable or in short supply (a common occurrence in Neonatal Intensive Care Units), the World Health Organization and the American Academy of Pediatrics recommend the use of donor milk (DM) as the best alternative [1,3]. Milk delivered to Human Milk Banks (HMBs) should be pasteurized so as to inactivate viral and bacterial agents [5]. The ideal pasteurization process should consist of a phase of rapid heating, followed by a phase in which the temperature is maintained constant, and a final phase of rapid cooling. Currently, a pasteurization process at 62.5 °C for 30 min (the Holder pasteurization method, HoP) is recommended in all international guidelines for the constitution of HMBs [5,6]. Pasteurized milk is known to retain many beneficial and protective effects of HM. This method is thought to lead to a good compromise between the microbiological safety and nutritional/biological quality of DM [7,8,9,10,11].

Nonetheless, it is also well known that HoP affects some of the nutritional and biological properties of HM. Several studies have been performed to investigate the effects of Holder pasteurization on the nutritional and immunological properties of DM, but a comprehensive review and comparison of the related results is, to the best of the authors’ knowledge, not available in literature. Currently, the available data consist of reviews regarding the advantages of donor milk, but very few details are given on the effects of HoP on the nutrients of mother’s milk [12,13,14,15,16].

Thus, the present paper is aimed at reviewing the published papers, and at comparing the results related to the effects of HoP on the biological and nutritive components of DM.

## 2. Search Methodology

The literature review was performed by conducting electronic searches of MEDLINE, EMBASE, CINHAL and the Cochrane Library. The electronic search used the following keywords and MeSH terms: (i) donor milk; (ii) banked milk; (iii) milk bank; (iv) milk banking; (v) (human milk OR donor milk) AND Holder pasteurization; (vi) (human milk OR donor milk) AND pasteurization; (vii) (human milk OR donor milk) AND storage; (viii) (human milk OR donor milk) AND heat treatment; (ix) (donor milk OR Holder pasteurization) AND protein; (x) (donor milk OR Holder pasteurization) AND enzyme; (xi) (donor milk OR Holder pasteurization) AND lipid; (xii) (donor milk OR Holder pasteurization) AND saccharide; (xiii) (donor milk OR Holder pasteurization) AND vitamin; (xiv) (donor milk OR Holder pasteurization) AND minerals; (xv) (donor milk OR Holder pasteurization) AND oxidative stress; (xvi) (donor milk OR Holder pasteurization) AND cytokine; (xvii) (donor milk OR Holder pasteurization) AND hormone; (xviii) (donor milk OR Holder pasteurization) AND growth factor; and (xix) (donor milk OR Holder pasteurization) AND immunoglobulin. No limits concerning the publication date were set.

Considering differences between the research protocols published to date, we focused the review on studies with an experimental design that:

- defined exactly the pasteurization method precisely (62.5–63 °C for 30 min); and

- compared the same samples of HM before and after the heat treatment.

In order to limit bias in the inclusion/exclusion process, the selection was made with the consensus of two authors (CP and MG).

## 3. Results

A total of 58 articles were found from a combination of the searches, but only 44 fulfilled all the inclusion criteria. Table 1 summarizes the technical details on the methodological aspects of the selected articles. Table 2, Table 3 and Table 4 provide an overview of the literature on the effects of HoP on different components of DM.

### 3.1. Energy Content

The first question that needs to be answered concerning the nutrition of all infants with pasteurized DM is: is this milk as nutritive as fresh HM? Only two studies have been found to have assessed the effects of HoP on the energy content of milk, with conflicting results. Ley et al. [17] reported that the HM energy content was not modified by pasteurization, whereas García-Lara et al. [18] found that the energy content was decreased significantly. The two studies used different approaches to calculate the energy content; in the first case [17], a direct measurement was performed by means of bomb calorimetry, whereas in the second study the energy content was calculated on the basis of the fat, protein and lactose contents, as measured by means of infrared spectroscopy [18]. The discrepancy in the results may be due to statistical artifacts rather than to real differences, since the percent decrease measured in the two studies was similar.

Overall, current evidence does not support the hypothesis of a relevant change in energy intake by feeding the infant with pasteurized rather than raw DM.

### 3.2. Nitrogenous Compounds

#### 3.2.1. Protein Content

The composition of the HM protein fraction varies from mother to mother, and changes during lactation. The protein content of term milk is estimated to be approximately 0.9 to 1.2 g/dL, while this value is higher for preterm milk [61]. The true protein content of HM is often overestimated, due to its high proportion of non-protein nitrogen [62]. In a study by Vieirà et al. [19], the average DM protein concentration, as assessed by means of an infrared analyzer, was significantly reduced by the HoP treatment, as was also found for colostrum [20]. Other studies, on the contrary, did not observe any significant change in protein content [17,21,22,23], even when the total nitrogen content was measured indirectly [18].

In conclusion, the majority of the examined reports indicates that HoP does not affect the protein content of DM. A statistically significant reduction was only found in two studies, although one of the studies, involving mature milk [19], reported a very slight reduction (−3.9%), similar to that found in the studies that claimed no effect on total protein content.

#### 3.2.2. Immunoglobulins (Igs)

The effect of HoP on the DM concentration of different classes of immunoglobulins has been investigated in several studies, the first being published in 1977 by Ford and colleagues [24].

IgAs and secretory IgAs (sIgAs) are the most extensively investigated classes, and almost all the published studies report a reduction following HoP. A significant reduction in IgAs following HoP was measured by means of Enzyme-Linked Immunosorbent Assays (ELISA) [25,26,27] and by means of Radial Immunodiffusion Assays (RIA) [28,29]. An IgA reduction was also reported in older studies [24,30], although no statistical significance was reached, perhaps due to weak experimental design. Ford et al. [24] analyzed a single pooled milk sample, while Evans et al. [30] used samples derived from overflow milk. The detrimental effect of HoP on IgAs was also confirmed on colostrum samples [20,31,32]. A reduction in protein bands recognized by means of anti-IgAs antibodies, although not quantified, was also reported [33]. Secretory IgAs, the dimeric forms of IgAs, were also found to have decreased following the pasteurization of DM [21,34,35], although not always significantly.

The other Ig classes were investigated in a smaller number of studies, whose results are partially contrasting, due to the extremely low Ig concentrations, and subsequent difficult detectability in the milk samples. However, the majority of the studies found some degree of reduction.

IgM concentrations were measured in mature DM after pasteurization, and were found to be significantly decreased [26,28,29], or even completely degraded [24]. The low resistance of IgMs to pasteurization has also been confirmed more recently on colostrum [20,31,32]. The same behavior has also been observed for IgG concentrations in both milk [26,28,29,30] and in colostrum [20,31,32]. The specific thermal resistance of different IgGs subclasses was also detailed: IgG1 were not affected by HoP, while IgG4 were reduced, and IgG2 and IgG3 were undetectable in both fresh and pasteurized milk samples [31].

As an overall conclusion, the results from the previous reports clearly indicate that one of the main detrimental effects of HoP is a reduction in all classes of immunoglobulins, probably due to the complex structure of these molecules.

#### 3.2.3. Lactoferrin and Lysozyme

The immunoprotective protein constituents of HM, with bacteriostatic properties, include lysozyme and lactoferrin [62].

Lactoferrin is an iron-binding protein that reduces the availability of the free iron required by iron-dependent pathogens, and therefore is able to inhibit their growth. Moreover, it can disrupt the bacterial cell membrane by binding to the lipid-A portion of lipopolysaccharides on the bacterial cell surface [63]. Lactoferrin has been investigated in several studies, using different techniques (ELISA, RIA, monospecific antisera) [24,29,30,31,32], all of which report a reduction in its concentration, with a percentage ranging from 35% to 90%, but the reduction was only reported as significant by Christen et al. [35]. Additionally, a reduction in the lactoferrin-containing band, by means of a protein electrophoresis semi-quantitative method, was reported [33]. Since the bactericidal activity of lactoferrin is maintained by bactericidal peptides that form during its digestion, it is possible that part of the activity is still retained in pasteurized HM despite reduction of the protein [62]. One recent survey [64] conducted by means of non-reducing protein electrophoresis, has reported that lactoferrin aggregation, rather than degradation, occurs following DM pasteurization with HoP. Whether this aggregation causes a decrease in lactoferrin bactericidal activity, is still not known.

A similar pattern emerges for lysozyme, whose concentration has been found to be reduced after pasteurization in several studies, with a percentage ranging from 20% to 85%. The biological activity of lysozyme was tested by Ford et al. [24], who did not find any significant difference. A significant reduction in lysozyme activity after HoP was found by other authors [25,36], even in colostrum [32]. It should be pointed out that, in those works, the lysozyme activity was always determined using a *Micrococcus lysodeikticus-*based turbidimetric assay, which measures to what extent bacterial growth is prevented by the addition of lysozyme-containing samples. Since DM is a complex mixture of several anti-bacterial enzymes and factors, it is not possible to discriminate if the reduction is due only to a decrease in lysozyme concentration.

#### 3.2.4. Other Enzymes

Owing to their different responses to heating, enzymes and their activity are commonly considered as markers for assessment of thermal treatments. Some of these enzymes (such as lactoperoxidase and alkaline phosphatase) are considered technological markers for pasteurization in bovine milk. Data on lactoperoxidase are scarce for HM, since its concentration is below the detection limit of commercial kits [24,32]. Alkaline phosphatase has also been found to be completely inactivated by HoP [21], as commonly found in bovine milk.

Because of the compensatory function of several HM enzymes for nutrient digestion in newborns [65], some researchers have focused on the activity of lipase and amylase milk enzymes. A complete degradation (both in concentration and enzymatic activity) has been found for lipoprotein lipase and bile salt dependent lipase [21,33,37], while amylase activity was partly retained [37]. The clinical relevance of variations in the enzymatic activity of these proteins still has to be investigated. In particular, the hypothesis of a reduction in nutrient absorption through feeding of pasteurized rather than raw HM, especially as far as lipid digestion is concerned, cannot be ruled out.

#### 3.2.5. Cytokines

Although cytokines are immunomodulatory components, it appears that most of those found in HM are anti-inflammatory, thereby possibly lessening the effect of infections [62]. The effect of pasteurization on several cytokines has been evaluated in colostrum [31]. Interleukin (IL)1β, IL2, IL4, IL5, IL6, IL8, IL10, IL12, IL13, IL17, Interferon (IFN)-γ, the Tumor Necrosis Factor (TNF)-α and Monocyte Chemotactic Protein (MCP)-1 were not significantly affected by the process. Rather remarkably, IL7 increased significantly after pasteurization, perhaps due to its release from cellular and/or fat compartments into the aqueous fraction, while Macrophage Inflammatory Protein-1β (MIP-1β) was significantly reduced [31]. IL2, IL4, IL5, IL12 and IL13 were unaffected even in pasteurized mature DM [38], while IL10 was decreased [38,39,40], as were IL1β [38], IFN-γ [39], IL6 [39] and TNF-α [38,39]. A significant increase in IL8, following HoP in mature DM, was also found [38,39].

In short, different degrees of thermal resistance were found for different cytokines, and the biological relevance of this altered balance in specific situations still remains to be addressed.

#### 3.2.6. Growth Factors

To date, only a few studies have evaluated the variation of growth factors (GF) in human milk after pasteurization, and each one has focused on one specific GF.

Transforming GF (TGF)-β2 was found to be stable in colostrum [31]. Epidermal GF (EGF) [40,41] and TGF-β1 [40] showed no difference before and after HoP. Heparin-Binding Epidermal-like GF (HB-EGF) was unaffected by heating, whereas Hepatocyte GF (HGF) was reduced to a great extent [38]. Insulin-like GF (IGF)-1 and 2, as well as IGF binding proteins 2 and 3, were reduced to a variable extent by pasteurization [41]. Granulocyte-Macrophage Colony-Stimulating Factor (GM-CSF) concentrations increased significantly after pasteurization, while the Granulocyte-Colony Stimulating Factor (GCSF) was not detected in any sample, before or after HoP, either in colostrum [31], or in mature milk [38].

Once again, as in the case of cytokines, the low number of studies to date, and the relevant variability between different growth factors, does not allow any conclusion on the effect of HoP to be generalized.

#### 3.2.7. Amino Acids

The DM amino acid composition was investigated in two studies, one [42] focusing only on sulfur amino acids (cysteine and methionine), and a few free amino acids. The authors reported that no significant modification was caused by HoP, with the exception of an increase in free glutamine [42]. In another study [43], the amino acid profile of pre- and post-pasteurization DM was found to be significantly different for arginine and leucine, to increase following HoP, and to decrease for aspartate. Nevertheless, the biological relevance of these variations was reported to be low, since the differences, although significant, were small.

The available lysine content, which is considered a nutritional marker, as lysine residues are targeted by proteases during digestion, was evaluated in two studies, with discordant results: Silvestre et al. [22] showed a 30% reduction (statistically significant) using a fluorimetric method, while Baro et al. [33] observed an increase in the concentration of this amino acid, measured by means of *o*-phthaldialdehyde. In this case, the discordant results may partially be due to the different experimental designs of the two studies, one of which simulated HoP [22], and the other used a commercial Holder pasteurizer [33].

### 3.3. Hormones

Insulin, adiponectin [17] and erythropoietin [40] concentrations were all reported to be decreased significantly after HoP, although the paucity of the reports does not allow any conclusion on the issue to be generalized.

### 3.4. Vitamins

The evidence on the effect of HoP on HM vitamins is contrasting. Folacin and vitamin B12 were evaluated in different studies with conflicting results, probably due to the significant instability of vitamins and to the variability between the study sampling and analysis methods of the studies. Ford et al. [24] found an important reduction in the capacity of DM to bind added vitamins after HoP, as measured by adding excess cyanocobalamin (vitamin B12 analog), while other studies [21,44] did not find any significant difference for vitamin B12 analyzed by means of a chemiluminescence immunoassay and a competitive protein binding assay. Stability of riboflavin, biotin, and the total pantothenic acid contents (vitamin B5) has also been observed [45]. A non-significant decrease was found for folacin (vitamin B9) after HoP [21,24,45]. Van Zoeren-Grobben et al. on the other hand, found a 30% reduction in folacin as a consequence of HoP, and a significant 15% reduction in vitamin B6 [44].

While the vitamin C concentration was unambiguously reported to be reduced by HoP [44,46,47], fat-soluble vitamins showed different responses to pasteurization. Vitamin D was unaffected [44]. In some reports, vitamin A was found to be reduced [48,49], but other authors found it stable [23,44], while tocopherols (vitamin E) were found not to be affected in [44,46], but reduced in [39,47].

To summarize, the available data seem to confirm a higher heat sensitivity for water soluble vitamins, and in particular for vitamin C, which is known to be highly susceptible to several technological treatments, including freezing and refrigeration [66], while most of the studies seem to indicate a higher retention of fat soluble vitamins (A, D, and partially E).

### 3.5. Zinc

Zinc levels have not resulted not to be significantly affected by HoP; however, a variation in the distribution of zinc was observed, with a significant increase in the fat fraction and a decrease in whey, possibly as a result of the denaturation of zinc binding proteins, thereby indicating possible consequences on zinc bioavailability to the infant [23].

### 3.6. Lipids

The total lipid content was evaluated in several studies: a significant reduction was found following HoP, using infrared analyzers [17,18,19], which, however, do not directly measure the fat content. Other authors have not found significant differences, when using different analysis techniques [23,37,50,51]. The total fatty acid profile was always found to be unaffected by pasteurization [37,39,43,47,50,52], with the exception of one study, which reported slight changes in the relative composition of medium-chain fatty acids (MCFAs) [38], and one of which reported a small decrease in 18:3 fatty acid [53]. Lepri et al. [51] found a more than two-fold free fatty acid content increase after pasteurization. A potential increase in the DM free fatty acid fraction following HoP, provided it does not cause off-flavors, may not be undesirable, as increased free fatty acids are known to be more readily absorbed in the digestive system, thus resulting in a possibly increased nutritive potential.

### 3.7. Saccharides

Several studies have evaluated the effect of HoP on lactose in mature milk and colostrum. In all cases, and using different analytical techniques, no significant difference was found before and after HoP [18,19,23,31,54]. The myoinositol levels were not affected by HoP either [31,54], while glucose was found to be increased [17], stable [31] and reduced [54], although all of the reported variations were low.

Glycosaminoglycans and oligosaccharides have recently been investigated in preterm milk, and no variation after the pasteurization process was observed [55,56]. These human milk glycans have been demonstrated to influence the health of newborns, since they possess specific biological properties, such as an anti-infective role, anti-oxidant functions and prebiotic effects [67].

In short, the evidence to date points toward the stability of DM saccharides during pasteurization by HoP, both as free molecules and as part of biologically active compounds, such as glycosaminoglycans and oligosaccharides.

### 3.8. Indicators of Thermal Treatment

Lactulose, a disaccharide formed through the isomerization of lactose, and furosine, an intermediate of the Maillard reaction, have been searched for in DM, since they are used in the food industry to differentiate between different kinds of heat-treated bovine milk, depending on the intensity of the heat treatment applied. Since HM has more than double the lactose content than bovine milk, lactulose formation has been found to be triggered more by pasteurization than is normally found for pasteurized bovine milk, in both milk [67] and colostrum [31]. Furosine was only found to have formed following pasteurization in colostrum samples [31], while it was not found in any milk sample [67].

Contador et al. [57] have very recently published a detailed analysis of volatile compounds in fresh and pasteurized HM, which supports the idea of some thermal-induced modification during HoP. The preservation of the original volatile compounds in HM is important, since they can negatively affect its quality, and indicate that undesired reactions have taken place during the treatment (e.g., lipid oxidation, Maillard reaction, etc.). A significant change in many volatiles was detected after HoP in DM; some of these volatiles (aldehydes, furans and pyrans) are undesired, and considered an index of thermal degradation.

### 3.9. Oxidative Stress Markers

The oxidative balance of raw and pasteurized HM was assessed by measuring both the accumulation of oxidants and the activity of oxidant scavengers. Malondialdehyde concentrations were not found to be significantly affected by HoP but, on the other hand, glutathione concentrations, glutathione peroxidase activity and Total Antioxidant Capacity were significantly reduced, thus indicating a decrease in the oxidative scavenging capacity of HM [58]. In a more recent assay [59], no change in malondialdehyde concentration, total antioxidant capacity (as measured by means of Oxygen Radical Absorbance Capacity assay), or hexanal concentration has been found, thus indicating no lipid oxidation and no decrease in oxidant scavengers.

### 3.10. Organic Acids

Only one study has assessed the l-lactate content of HM, following HoP, using a biosensor based on an immobilized lactate oxidase enzyme. The results showed a significant increase, probably due to the release from interaction with other substances [51].

### 3.11. Recently Published Research

One study has recently highlighted an increase in the nucleotide monophosphate content following pasteurization, measured by means of mass spectrometry. These compounds are considered as immune-enhancers and seem to play a role as sleep-inducers, so their increase in HM may be a positive consequence of HoP [60]. Nevertheless, further evidence is needed before any claim on the issue could be formulated.

## 4. Discussion and Conclusions

The present review shows a significant variability in the data reported in the scientific literature concerning the effects of HoP on the biological components of HM.

A possible explanation for this variability may be the heterogeneity of the test protocols applied in the studies (e.g., in terms of sample origin, storage conditions or analysis methods). Another important source of variability is represented by the fact that the Holder pasteurization of donor milk is often simulated on small aliquots, rather than being performed following HMB-implemented protocols. Moreover, modern pasteurizers require significantly less time for heating and cooling than older ones, thus changing the kinetics of the thermal response for heat-sensible compounds. Additionally, it appears that some biochemical patterns were investigated more extensively than others, while some other milk components were not considered at all.

The results of the review can be summarized as follows. Saccharides are not significantly affected by the heat treatment, as either free molecules or as part of biologically active compounds. The total lipid content is preserved by HoP, as is its fatty acid composition. This finding is of paramount importance since it suggests that pasteurization is able to preserve both the nutritional and biological properties relevant to the development of the central nervous system associated with some of these fatty acids. Consistently, fat soluble vitamins also seem to be unaffected, while water soluble vitamins, and vitamin C in particular, are generally reported as significantly decreased. The results concerning specific biologically active molecules (such as cytokines and growth factors) remain uncertain, due to the vast number of different compounds analyzed in each study, and to the paucity of comparable results.

Proteins are more significantly affected by HoP. In fact, specific proteins with significant immunologic and anti-infective action (such as immunoglobulins and lactoferrin) are reduced by pasteurization. A substantial reduction in the enzymatic activity has also been observed. The review thus confirms the main concerns about Holder pasteurization of HM, and the need for future strategies to prevent and/or limit DM protein degradation. The available data confirm that HoP affects several HM components to variable degrees, even though it is rather difficult to quantify the degradation level. Nonetheless, clinical practices demonstrate that many beneficial properties of human milk remain, even after pasteurization.

Future studies should be aimed at confirming the currently available data by investigating more reproducible analytic settings, while avoiding the introduction of potential biases, in order to understand the real effects of pasteurization on mother’s milk. Moreover, further studies should be focused on new pasteurization techniques in order to improve the biological quality and safety of DM.

## Figures and Tables

**Table 1:** Materials and Methods of the different studies included in the survey.

Ref *	Preterm/Term	Phase of Lactation	Expression Method	Status	Pre-Pasteurization Storage	Pasteurization Equipment	Sample Size	Analytical Method °
[17]	N/A	Mature	N/A	Frozen	−20 °C up to 6 months; thawing in a water bath at 37.5 °C	Sterifeed: pre-heated water bath (63.2 °C); 62.5 °C for 30′; cooling in cold water bath	17 pools—4 donors each	Adiponectine: RIA
								Insulin: electrochemiluminescence immunoassay
								Total fat: creamatocrit
								Total protein: BCA
								Total energy: bomb calorimetry
								Glucose: enzymatic method
[18]	Preterm and Term	Mature	Hand or electric/manual pump	Frozen	−20 °C until processing; thawing and heating to 40 °C using a thermostatic bath	62.5 °C for 30′; cooling to <4 °C in stirred thermostatic baths	34 samples—28 donors	Infrared Analyzer (MIRIS)
[19]	N/A	N/A	Hand or electric/manual pump	Fresh	No	62.5 °C for 30′	57 samples	Infrared Analyzer (Milko-scan Minor)
[20]	Preterm and Term	Colostrum	Hand	Fresh	No	62.5 °C for 30′	36 samples: <32 weeks; 32 samples: 32–36 weeks; 33 samples: >36 weeks	Total protein: refraction index
								Lysozyme: lysoplate method
								Immunoglobulins: RIA
[21]	Term	N/A	N/A	Fresh	No	LABU-Muttermilch pasteurizer: 62.5 °C for 30′	4 Samples—2 CMV-positive and 2 CMV-negative donors	Total protein, alkaline phosphatase and lipase activity: Hitachi 917 Automatic Analyzer
								Folic acid, Vitamin B12: chemiluminescence immunoassays
								sIgA and lysozyme: RIA
[22]	N/A	Mature	Electric pump	Fresh	No	VLM exchangeable HBV-Q-16-16: 63 °C for 30'	30 samples—30 donors	Lysine content: fluorimetry
								Total protein: Lowry method
[23]	Term	Mature	Hand or electric/manual pump. Occasional drip milk	Frozen	−20 °C up to 15 days; thawing in a microwave oven	62.5 °C for 30′; cooling by ice-cold water for 10′	15 samples from individual mother or pool (5 donors)	Total fat: crematocrit
								Total protein: Lowry method
								Lactose: picric acid method
								Vitamin A: HPLC
								Zinc: Atomic absorption spectrometry
[24]	N/A	Mature	N/A	Fresh	Refrigeration at 4 °C for 1 to 2 days; centrifugation at 2 °C for 1 h; −30 °C until testing	62.5 °C for 30′	1 pool—25 donors	Immunoglobulins and lactoferrin: RIA
								Vitamins: labeled cyanocobalamin, separation of free and protein-bound vitamins by gel filtration
[25]	N/A	N/A	N/A	Frozen	−40 °C until analysis	62.5 °C for 30′	1 pool—10 donors	Total protein: BCA
								Immunoglobulins: ELISA
								Lysozyme activity: *Micrococcus lysodeikticus* turbidimetric assay
[26]	N/A	Mature	N/A	Fresh	Refrigeration	62.5° C for 30′ in stirred water bath	2 pools—5 and 6 donors	Immunoglobulins: ELISA
[27]	Term	Mature	Electric pump	Fresh	–80 °C until the analysis	62.5 °C for 30′	10 samples—10 donors	Immunoglobulins: ELISA
[28]	N/A	Mature	N/A	Fresh	No	63 °C for 30′	23 samples	Immunoglobulins: RIA
[29]	Term	Colostrum, transitional and mature	Manual or pump	Fresh	Refrigeration in ice	62.5 °C for 30′	5 samples—89 donors	Immunoglobulins: RIA
								Lactoferrin: Laurell method
[30]	N/A	N/A	Overflow milk	Fresh	Refrigeration up to 48 h	62.5 °C for 30′	16 samples	Electroimmunoassay against monospecific antiserum
[31]	N/A	Colostrum and mature	HMB protocol	Fresh	N/A	62.5 °C for 30′	10 colostrum and 8 mature milk	Furosine: HPLC
								Carbohydrates: gas chromatography
								Cytokines: ELISA
								Immunoglobulins: ELISA
[32]	N/A	Colostrum	Hand or electric pump	Fresh	−20 °C until analysis	62.5 °C for 30′	1 pool—11 donors	Immunoglobulins: ELISA
								Lysozyme activity: *Micrococcus lysodeikticus* turbidimetric assay
								Lactoperoxidase activity: ABTS assay
[33]	Term	Transitional	Electric pump	Fresh	No	Metallarredinox: 62.5 °C for 30′	1 pool—4 donors	IgA, lactoferrin: SDS-PAGE, Western Blot and mass spectrometry
								Lipase activity: p-nitrophenol assay
								Available lysine: OPA method
[34]	N/A	N/A	N/A	Fresh	N/A	3 devices: Sterifeed–Saurin–Carag: 62.5 °C for 30′	10 samples for Sterifeed; 6 samples for Saurin; 6 samples for Carag	Lysozyme, sIgA and lactoferrin: ELISA
[35]	N/A	N/A	N/A	Frozen	−20 °C until analysis	62.5 °C for 30′	10 samples—10 donors	Lysozyme, sIgA and lactoferrin: ELISA
[36]	N/A	N/A	Drip milk	Fresh	No	Semi-automated Holder pasteurizer	1 pool—20 donors	IgA: electroimmunoassay
								Lysozyme activity: *Micrococcus lysodeikticus* turbidimetric assay
[37]	N/A	Mature	Electric pump	Frozen and fresh	–70 °C; thawing in cool water	62.5 °C for 30′ in stirred water bath	6 samples: 3 pools from HMB and 3 samples from 3 donors	Total fat: gravimetry
								Fatty acids: gas chromatography
								Lipase activity: triglyceride emulsion
								Amylase: ELISA
[38]	N/A	Mature	N/A	Frozen	4 °C overnight; thawing at 37 °C in water bath, gently mixed	Sterifeed: pre-heated water bath (63.2 °C); 62.5 °C for 30′; cooling in cold water bath	17 pools—4 donors each	Cytokines and growth factors: ELISA
								Fatty acids: gas chromatography
[39]	N/A	Mature	N/A	Fresh	Refrigeration until analysis	62.5 °C for 30′ in water bath under constant agitation	1 pool—6 donors	Tocopherol: HPLC
								Fatty acids: gas chromatography
								Cytokines: ELISA
[40]	Term	Mature	Hand or electric pump	Fresh	No	62.5 °C for 30′	17 samples	Cytokines and growth factors: ELISA
[41]	Preterm and Term	Transitional and mature	N/A	Frozen	−20 °C until analysis	LABU-Muttermilch pasteurizer: 63 °C for 30′	51 samples—28 donors	IGF and IGFBP: RIA
								EGF: ELISA
[42]	Term	Mature	N/A	Fresh	No	62.5 °C for 30′	13 samples—13 donors	Free amino acids: HPLC
[43]	Preterm and Term	Colostrum, transitional and mature	N/A	Frozen	Thawed overnight	ACE pasteurizer: 62.5 °C for 30′	39 samples—3–4 donors each	Fatty acids: gas chromatography
								Free amino acids: Amino acid Analyzer
[44]	Term	Mature	Manual pump	Frozen	Refrigeration max 4 h; −20 °C up to 3 weeks	62.5 °C for 30′	5 samples—9 pools	Vitamins: HPLC
[45]	Term	Mature	Hand or pump	Fresh	Refrigeration	62.5 °C for 30′	5 each—89 donors	Vitamins: HPLC
[46]	N/A	Mature	Electric pump	Frozen	−80 °C until analysis	62.5 °C for 30′	10 samples—10 donors	Vitamin C, Tocopherols: HPLC
								Fatty acids: gas chromatography
[47]	N/A	Mature	Manual pump	Fresh	No	62.5 °C for 30′	1 pool—10 donors	Vitamin C, Tocopherols: HPLC
								Fatty acids: gas chromatography
[48]	N/A	N/A	N/A	Frozen	Frozen	63 °C for 30′	50 samples—50 donors	Vitamin A, beta catotene: HPLC
[49]	N/A	Colostrum, transitional and mature	N/A	Frozen	Thawing in ice-filled plastic container for 15′	63 °C for 30′	60 samples	Vitamin A: gas chromatography
[50]	Preterm and Term	Transitional	Electric pump	Fresh	No	62.5 °C for 30′; cooling in running water	12 samples—12 donors	Fat content: gravimetry
								Fatty acids: gas chromatography
[51]	Term	N/A	Hand	Fresh	No	62.5 °C for 30′	1 pool—16 donors	Fatty acids: gas chromatography
								L-lactate in milk: enzymatic biosensor
[52]	N/A	N/A	N/A	Fresh	No	63 °C for 30′	3 samples—3 donors	Total fat and fatty acids: gas chromatography, Infrared spectroscopy and NMR
[53]	N/A	Transitional	N/A	Fresh	No	62.5 °C for 30′; cooling in running water	7 samples—1 donor	Fatty acids: gas chromatography
[54]	N/A	N/A	Electric/manual pump	Fresh	N/A	62.5 °C for 30′; cooling in stirred ice-cold water bath	21 samples—21 donors	Furosine: HPLC
[55]	Preterm	N/A	Electric pump	Fresh	No	Sterifeed: 62.5 °C for 30′	10 samples—10 donors	Oligosaccharides: HPLC
[56]	Preterm	N/A	Electric pump	Fresh	No	Sterifeed: 62.5 °C for 30’	9 samples—9 donors	Glycosaminoglycans: HPLC
								Carbohydrates: gas chromatography
[57]	N/A	Mature	N/A	Fresh	Refrigeration	62.5 °C for 30′ in stirred water bath	1 pool—8 donors	Volatile compounds: gas chromatography—mass spectrometry
[58]	Term	N/A	Electric pump	Fresh	No	62.5 °C for 30′	31 samples—31 donors	MDA and GSH: HPLC
								GPx activity: Lawrence and Burk method
								ToAC: commercial kit
[59]	N/A	Mature	Hand	Frozen	−80 °C up to 2 weeks	62.5 °C for 30′	30 samples—10 donors	Total fat : creamatocrit
								Fatty acids and volatiles: gas chromatography
								MDA: TBARS
								Tocopherols and ascorbic acid: HPLC
								ToAC: ORAC
[60]	N/A	Mature	Hand	Frozen	−40 °C in HMB; −18 °C in lab	62.5 °C for 30′	1 pool—5 donors	Free nucleotide monophosphates: capillary electrophoresis—mass spectrometry

* Ref: number in reference list; ° RIA: Radioimmunoassay; ELISA: enzyme-linked immunosorbent assay; HPLC: high-performance liquid chromatography; BCA: bicinchoninic acid assay; ABTS: 2,2′-azinobis-(3-ethylbenzothiazoline-6-sulfonic acid); SDS-PAGE: Sodium Dodecyl Sulphate-PolyAcrylamide Gel Electrophoresis; OPA: o-phthaldialdeyde; NMR: Nuclear Magnetic Resonance; MDA: malondialdehyde; GSH: reduced glutathione; GPx: glutathione peroxidase; ToAC: total antioxidant capacity; TBARS: thiobarbituric acid reactive species; ORAC: Oxygen Radical Absorbance Capacity.

**Table 2:** DM components not affected by Holder Pasteurization (HoP) (consensus on results).

Components	Reference
Total Nitrogen Content	[18]
Cytokines	
IL2, IL4, IL5, IL13	[31,38]
IL12p70	[31,38,39]
IL17	[31,39]
Growth Factors	
EGF	[40,41]
TGFβ1	[40]
TGFβ2	[31]
MCP-1	[31]
Amino acids	
Free amino acids	[43]
Taurine, methionine, cystine, glutamate	[42,43]
Vitamins	
D, E, B2	[44]
B5, Biotin, B3	[45]
B12	[21,24,44,45]
*Zinc*	[23]
Lipids	
Polyunsaturated fatty acid *n*3	
20:5	[37,50]
22:5	[38,47,50]
22:6	[37,39,43,47,50]
Polyunsaturated fatty acid *n*6	
18:2	[37,38,39,43,47,50,51,52,53]
18:3	[39,43,47,50]
20:2	[47,50]
20:3	[39,47,50]
20:4	[37,38,39,43,47]
22:4	[37,47,50]
22:5	[38,47]
Monounsaturated fatty acid	
14:1	[37,39,43,47,50]
15:1	[47,50,52]
16:1	[37,38,39,43,47,50,51,52,53]
17:1; 22:1	[47,50]
20:1	[39,47,50]
24:1	[37,47,50]
Saturated fatty acid	
10:0, 16:0	[37,38,39,43,47,50,51,52]
15:0	[39,47,50,51]
17:0	[39,47,50]
20:0	[39,47,50,52]
21:0	[47]
22:0	[47,50]
24:0	[37,50]
Saccharides	
Oligosaccharides	[55]
Glycosaminoglycans	[56]
Myoinositol	[31,54]
Lactose	[18,19,23,31,54]
Oxidative Stress Markers	
Malondialdehyde	[58,59]
ORAC and Hexanal	[59]

**Table 3:** DM components affected significantly by HoP (consensus on results).

Components	Effect of HoP	References
Immunoglobulins		
IgG4	Reduction (% not reported)	[31]
Enzymes		
Lipase	Complete loss	[21,33,37]
Alkaline phosphatase	Complete loss	[21]
Amylase	Reduction (15%)	[37]
Cytokines		
IL7	Increase (% not reported)	[31]
MIP-1β	Reduction (% not reported)	[31]
MCAF/MCP-1	Reduction (% not reported)	[39]
Growth Factors		
IGF1, IGF2, IGFBP2, IGFBP3	Reduction (% not reported)	[41]
EPO	Reduction (% not reported)	[40]
HB-EGF, HGF	Reduction (% not reported)	[38]
GM-CSF	Increase (% not reported)	[31]
Hormones		
Insulin, Adiponectin	Reduction (% not reported)	[17]
Free amino acids		
Arginine, leucine	Increase	[43]
Aspartate	Reduction (% not reported)	[43]
Glutamine	Increase (% not reported)	[42]
Vitamins		
Ascorbic + Dehydroascorbic	Reduction (12%)	[47]
Ascorbic Acid	Reduction (16.2%–26%)	[46,47]
B6	Reduction (15%)	[44,46]
Oxidative Stress Markers		
Glutathione, Glutathione peroxidase activity, Total antioxidant capacity	Reduction (% not reported)	[58]
Lactulose	Increase (% not reported)/(Not detected in all samples)	[31,54]
Nucleotide monophosphate content	Increase (% not reported)	[60]

**Table 4:** DM components affected by HoP (discordant results).

Components	Effects of HoP	References
Total Protein Content	Reduction (% not reported)	Significant: [17,19,20]
		Not significant: [21,22,23,37]
Immunoglobulins		
IgA	Reduction (20%–62%)	Significant: [25,28,29,31,32]
		Not significant: [20,24,26,27,30,35]
sIgA	Reduction (20%–50%)	Significant: [35]
		Not significant: [21,34]
IgM	Reduction (50%–100%)	Significant: [28,29,31,32]
		Not significant: [20,24,26]
IgG	Reduction (23%–100%)	Significant: [32]
		Not significant: [20,24,26,28,29,30]
Lactoferrin	Reduction (35%–90%)	Significant: [35]
		Not significant: [24,29,30,33,34]
Lysozyme		
concentration	Reduction (20%–69%)	Significant: [34] (Sterifeed and Carag), [35]
		Not significant: [20,21,30,34] (Saurin)
activity	Reduction (% not reported)	Significant: [25,32]
		Not significant: [24]
Cytokines		
IL1beta, IL6	Reduction (% not reported)	Significant: [39]
		Not significant: [31]
IL8	Increase (% not reported)	Significant: [38,39,40]
		Not significant [31]
IL10	Reduction (% not reported)	Significant: [38,39]
		Not significant: [31]
TNF alfa	Reduction (% not reported)	Significant: [38,39]
		Not significant: [31]
INF gamma	Reduction (% not reported)	Significant: [38]
		Not significant: [31,39]
Vitamins		
A	Increase (% not reported)/reduction (34%)	Significant: [49]
		Not significant: [23,44,48]
Folacin	Reduction (10%–30%)	Significant: [44]
		Not significant: [21,24,29]
C	Reduction (19.9%–36%)	Significant: [44,46]
		Not significant: [29]
alfa- and gamma-Tocopherol	Reduction (12%–47%)	Significant: [39,47]
		Not significant: [46]
delta-Tocopherol	Reduction (% not reported)	Significant: [39]
		Not significant: [46]
Total fat content	Reduction (% not reported)/Increase (% not reported)	Significant: [17,18,19]
		Not significant: [23,37,50,51]
Polyunsaturated fatty acid *n*3		
18:3	Reduction (% not reported)	Significant: [53]
		Not significant: [38,39,47,50]
Monounsaturated fatty acid		
18:1	Increase/reduction (% not reported)	Significant: [38]
		Not significant: [39,47,50,53]
Saturated fatty acid		
14:0	Increase/reduction (% not reported)	Significant: [38]
		Not significant: [37,39,43,47,50,52,53]
12:0	Increase/reduction (% not reported)	Significant: [38,43]
		Not significant: [37,39,47,50,51,52]
18:0	Increase/reduction (% not reported)	Significant: [38]
		Not significant: (2015) [37,39,43,47,50,51,52]
Glucose	Reduction/Increase (% not reported)	Significant: [17] (increase), [54] (reduction)
		Not significant: [31]
